# Probiotics in Cancer

**DOI:** 10.3389/fonc.2021.638148

**Published:** 2021-03-12

**Authors:** Ke Lu, Shanwu Dong, Xiaoyan Wu, Runming Jin, Hongbo Chen

**Affiliations:** ^1^ Department of Pediatrics, Union Hospital, Tongji Medical College, Huazhong University of Science and Technology, Wuhan, China; ^2^ Department of Pediatrics, Wuhan Fourth Hospital, Wuhan, China; ^3^ Department of Pediatrics, Puai Hospital, Tongji Medical College, Huazhong University of Science and Technology, Wuhan, China

**Keywords:** probiotics, cancer, safety, clinical trials, treatment

## Abstract

In recent years, the consumption of over-the-counter probiotics to promote health has grown rapidly worldwide and become an independent industry. In medicine, various studies have demonstrated that probiotics can help improve the immune system and intestinal health. They are usually safe, but in some rare cases, they may cause concerning adverse reactions. Although the use of probiotics has been widely popularized in the public, the results of many probiotic clinical trials are contradictory. Particularly in cancer patients, the feasibility of probiotic management providing benefits by targeting cancer and lessening anticancer side effects requires further investigation. This review summarizes the interactions between probiotics and the host as well as current knowledge on the pros and cons of utilizing probiotics in cancer patients.

## Introduction

In the human intestine, there are more than 100 trillion symbiotic bacteria, far exceeding the number of host cells, which together constitute the intestinal flora (1). They affect multiple functions of the host, and the stability of the intestinal flora is essential for preventing pathogen infection and disease (2). The history of human consumption of probiotics can be traced back as early as 1907 (3). After more than a century of screening, lactic acid bacteria and Bifidobacteria have dominated the market. Among them, Bifidobacterium (adolescentis, animalis, bifidum, breve, and longum) and Lactobacillus (acidophilus, casei, fermentum, gasseri, johnsonii, paracasei, plantarum, rhamnosus, and salivarius) are the most commonly used species on the market (3). At the same time, several other strains seem promising for human health, such as Roseburia spp., Akkermansia spp., and Faecalibacterium spp., which are worthy of in-depth investigation (4).

In recent years, studies on the use of probiotics for the prevention and treatment of human diseases have been performed globally (1). At present, a variety of beneficial mechanisms have been identified, including regulating intestinal flora, enhancing intestinal barrier function, protecting intestinal epithelium from invasion by pathogens and strengthening immune function (5, 6).

**Figure 1:**
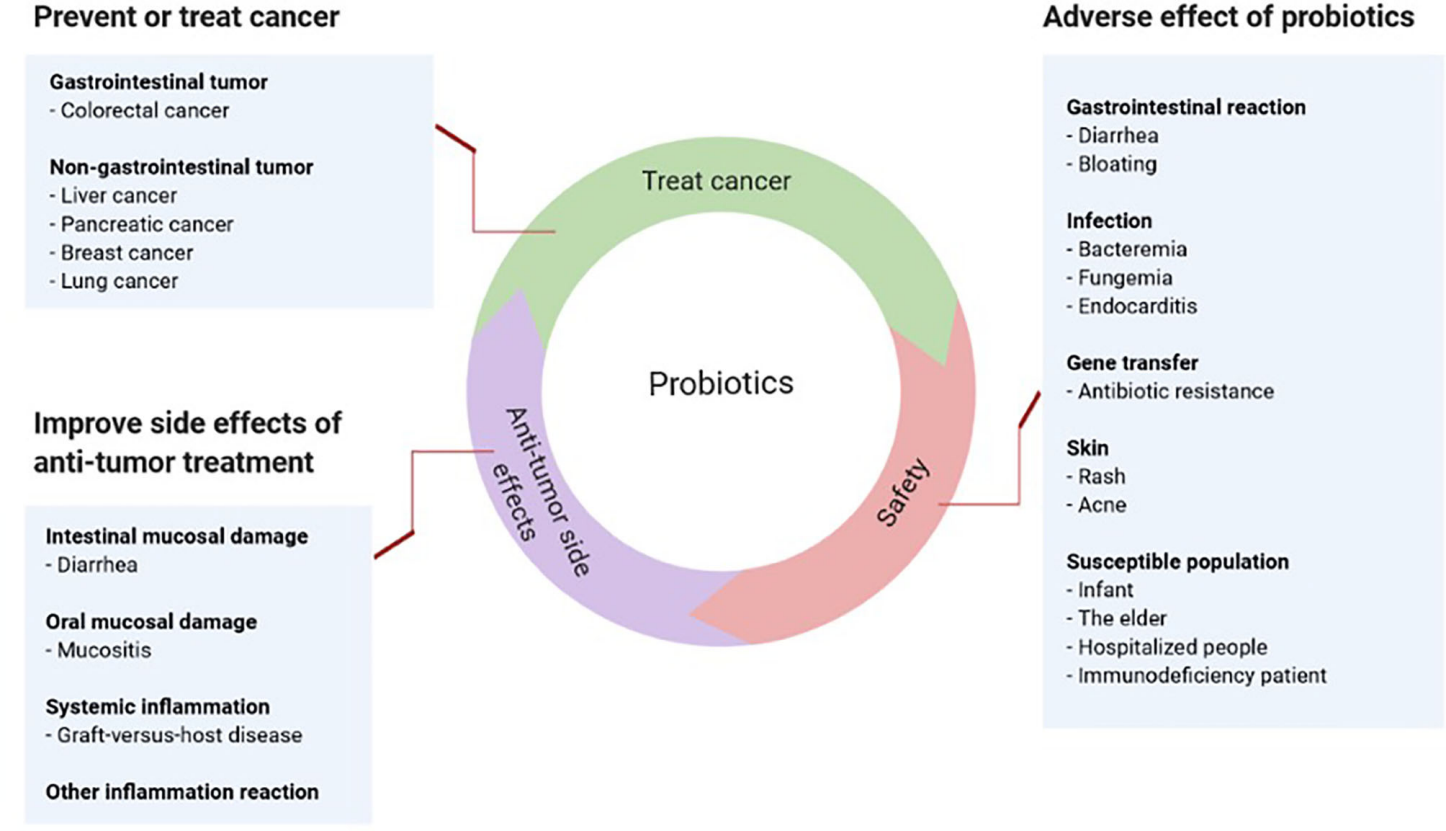
Pros and cons of probiotics in cancer.

Cancer patients have compromised immunity caused by primary diseases, chemotherapy and radiotherapy. The effects of probiotics in this population may differ from those of healthy people and raise several critical concerns (7). Therefore, this article reviews whether cancer patients can take probiotics as well as their pros and cons (**Figure 1**).

## The Effect of Probiotics on the Host

**Figure 2:**
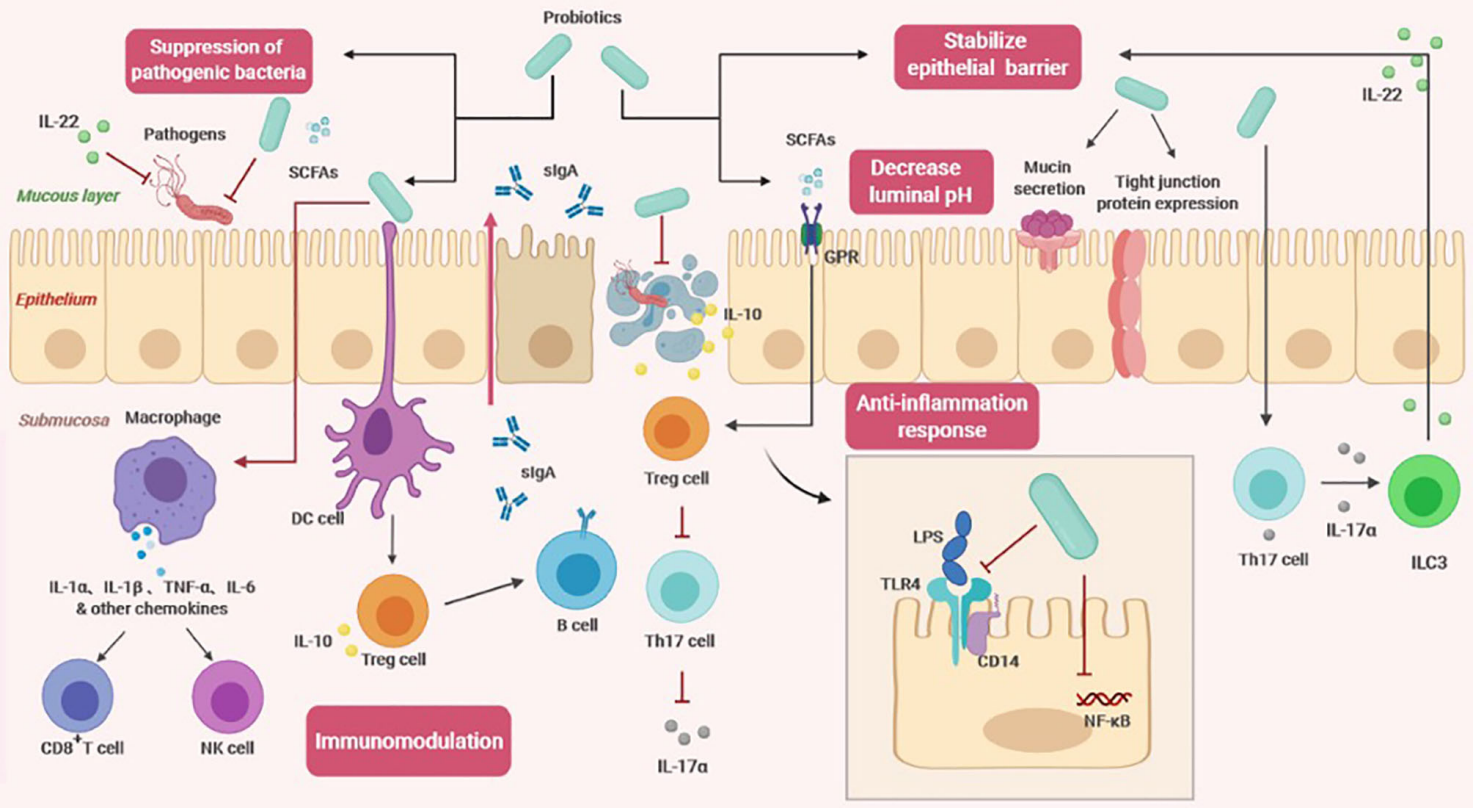
The effects of probiotics on the host (SCFAs, short-chain fatty acids; sIgA, soluble IgA; GPR, G protein coupled free fatty acid receptor; DC cell, dendritic cell; Treg cell, regulatory T cell; Th17, T helper cell 17; ILC3, Type 3 innate lymphocyte; NK cell, Natural killer cell; LPS, lipopolysaccharide; TLR4, Toll-like receptor 4; NF-κβ, nuclear factor-κB).

Studies have confirmed that probiotics can exert a variety of beneficial effects on the host. In addition, probiotic metabolites, such as short-chain fatty acids (SCFAs) and lactic acid, also play a significant role (4). Using forward chemical genetic screening, a recent study found that multiple probiotic metabolites modulate host physiology by activating G protein-coupled receptors (GPCRs) (8). Based on the contribution of probiotics to intestinal health, it is currently believed that the core benefit of probiotic management is to maintain healthy intestinal flora and support a healthy immune system through nonspecific and specific physiological effects, respectively (8) (**Figure 2**).

### Nonspecific Physiological Effects

Regulation of intestinal flora: probiotics can maintain a healthy balance of intestinal flora. By studying fecal specimens, it was found that supplementation with probiotics may increase the count of specific bacterial strains in healthy adults, suggesting that probiotics may cause changes in the total number, diversity and composition of intestinal flora (9). In the past, this has been used as an evaluation standard, but considering that fecal flora only reflect part of the intestinal flora information, a great deal of information is missing when evaluating fecal samples only (10). The closer the sampling site is to the end of the rectum, the less it reflects the structure of the upper flora. In a large-scale genomic analysis, fermented foods were indeed an important source of intestinal lactic acid bacteria, providing unprecedented evidence that food-derived probiotics are closely related to the composition of intestinal microorganisms (10).

Stabilizing the intestinal epithelial cell barrier: probiotics regulate the cytoskeleton to stabilize the mucosal barrier and promote mucin secretion to prevent the colonization of pathogens in the epithelium (11). They can induce expression and distribution of tight junction proteins (12). By sealing the top epithelium and endothelium, an increase in epithelial permeability and damage to the epithelial structure are prevented. Probiotics could also restore abnormal transepithelial resistance caused by pathogenic lipopolysaccharide (LPS), thereby reducing the inflammatory response and excessive apoptosis (12). In addition, certain probiotic strains regulate the polarization of T helper 17 (Th17) cells and effectively induce secretion of IL-17α, which triggers type 3 innate lymphocytes (ILC3s) to produce IL-22 (6). IL-22 is a key immune defense cytokine that plays an important role in maintaining intestinal homeostasis and promoting healing and tissue regeneration. Animal experiments have revealed that mice lacking these cytokines are prone to experimental colitis due to defects in defensin secretion and damaged epithelial tight junctions (13).

Inhibiting pathogens: There are primarily two distinct mechanisms of inhibiting pathogens. One belongs to the physical defense system. The infection of pathogens starts from colonization on the surface of the intestinal mucosa, causing tissue damage. When probiotics completely occupy the space of the intestinal wall, there is no available space for pathogens, and probiotics can further inhibit the adhesion of pathogenic bacteria by obtaining more nutrients (7). The other mechanism is related to the antagonistic properties of probiotics, which can reduce the microenvironment pH by producing SCFAs (11). Some studies have found that SCFAs are primarily produced by utilization of undigested carbohydrates by colon anaerobic bacteria, mainly acetic acid, propionic acid, and butyric acid. The high concentration of SCFAs that accumulate in the intestinal tract can quickly lower the pH (14). Compared to pathogens, probiotics are more able to adapt to lower pH environments and therefore have a better survival rate. In addition to changing the pH value, probiotics also antagonize pathogen adhesion and transport through other mechanisms (7). A new study showed that IL-22 derived from the intestinal flora regulated mucosal glycosylation modification, promoted the growth of the symbiotic bacterium Phascolarctobacterium, and competed with Clostridioides difficile for succinate, preventing Clostridioides difficile infection (15).

### Specific Physiological Effects

Immune regulation: Probiotics can regulate humoral immunity, innate immunity and cellular immunity through distinct mechanisms (11). Despite some commonalities between probiotic and pathogenic surface molecules, intestinal epithelial cells can perceive and distinguish between symbiotic and pathogenic bacteria through cytokine production and signal transduction (16). After probiotics come into contact with intestinal epithelial cells, host dendritic cells (DCs) accurately recognize probiotic surfaces and effector molecules through pattern recognition receptors and coreceptors and then present antigens to regulatory T cells (Tregs) after processing (17). The increase in the number of Tregs promotes the transformation of B cell antibody classes and the secretion of large amounts of sIgA (17). Recent studies have shown that in addition to T cell-dependent pathways, sIgA production is also regulated through T cell-independent pathways (18). This process is mediated by metabolite-sensing free fatty acid receptors (18). After SCFAs bind to fatty acid receptors, they induce dendritic cells to express class 1A acetaldehyde dehydrogenase (Aldh1a), which converts vitamin A into retinoid acid, thereby assisting in the production of sIgA (18). In addition, probiotics activate macrophages to secrete cytokines and subsequently activate host natural killer cells and cytotoxic T cells, which participate in the immune response to clear pathogens (16). SCFA-mediated G protein-coupled free fatty acid receptor 43 (GPR43) signaling also causes NLRP3 inflammasome activation and secretion of IL-18 to further limit pathogen invasion (19).

Anti-inflammatory response: There are reports of probiotics inducing both anti-inflammatory and pro-inflammatory responses. Although this may seem contradictory at first glance, it indicates that probiotics have an important balancing effect on intestinal homeostasis in different contexts (20). Through multiple signaling pathways, probiotics can regulate the expression of cytokines, chemokines, and antimicrobial peptides, including the nuclear factor-κB (NF-κβ) and mitogen-activated protein kinase (MAPK) pathways (16). The role of probiotics in the anti-inflammatory response is related to their ability to regulate Toll-like receptors (TLRs) and GPRs. Probiotics could stimulate negative regulatory factors (A20, Bcl-3, and MKP-1) to attenuate LPS-induced TLR4 activation (21). They can also inhibit binding of LPS to the CD14 receptor, reducing the overall activation of NF-κβ (22). After SCFAs bind to GPR, the regulatory function of Foxp3^+^ Treg cells is enhanced, increasing IL-10 production. Tregs recognize protection in various inflammatory diseases, so SCFA signaling reduce sensitivity to chronic inflammation (22). Another study indicated that GPR109A on the surface of dendritic cells and macrophages recognizes butyrate, promotes Treg development and inhibits proliferation of proinflammatory Th17 cells (19).

## Effects of the Host on Probiotics

It has been reported that the same strain has differential effects on host physiology. Distinct from medicines, the efficacy of probiotics varies greatly from individual to individual. Age, physical condition, intestinal microbial composition, colonization permission and diet of the host all contribute to the heterogeneity of the effect (23). In infants and young children whose immune function is not yet fully developed, during the first month after birth, the development of intestinal flora is essential for the balanced development of the baby’s immune system. Bifidobacterium in breast milk is not only noncytotoxic but also has good immunostimulatory ability, but there is insufficient evidence to show that supplementation with probiotics is beneficial to infant health (14). In an observational study, although probiotic supplementation increased infant sIgA response, the incidence of mucosa-related diseases was higher in early childhood (24). Compared to healthy adults, the beneficial effects of probiotic exposure in infancy were not only limited but were also related to increased infections later in life (24).

In cancer patients, after undergoing treatments, such as chemotherapy, radiotherapy or surgical eradication, underlying medical conditions, such as cachexia combined with treatment-related side effects, and the microenvironment are more complicated, and can directly lead to intestinal mucosal barrier destruction and immune system dysfunction. The above changes are not conducive to the colonization of beneficial probiotics in the colon (25). In individuals with colorectal cancer, a reduction in the number of probiotics was observed (26). Zmora, N. et al. found that host local intestinal microbes also played a central role in the colonization of probiotics, and the useful function of probiotics was dependent on the support of the intestinal flora (27). These results indicate that even if the probiotics used are beneficial, the colonization barrier will greatly affect the therapeutic effect. There is an urgent need to elucidate the effects of probiotics in specific populations, such as cancer patients.

The intestinal microecology is composed of intestinal flora, prebiotics and enteral nutrition, which complement one another. Therefore, probiotics need a suitable environment to function. A variety of foods has been added to maintain healthy flora (28). For example, fermentable carbohydrates support the colonization and growth of beneficial bacteria in the intestine (29). Dietary fiber stimulates the growth and activity of beneficial bacteria and can reduce stomach acid to protect probiotics, allowing them to pass smoothly into the intestine. Polyunsaturated fatty acids regulate the adhesion of probiotics (9). For cancer patients, in addition to individual factors, dietary difficulties and the occurrence of malnutrition accelerates the collapse of intestinal homeostasis caused by cancer. In this vicious cycle, the therapeutic effect of probiotics is greatly reduced (30).

## Probiotics to Prevent and Treat Cancer

The results of many *in vitro* studies have shown that probiotics have beneficial properties in regulating proliferation and apoptosis of cancer cells (31). For example, it has been demonstrated that in mouse colon cancer HGC-27 and human colon cancer Caco-2, DLD- 1, and HT-29 cells that Lactobacillus rhamnosus GG strain inhibits proliferation and induces apoptosis (32).

In preclinical experiments, potential antitumor products include probiotics and their metabolites, such as butyrate and pyridoxine. SCFAs are the energy source of colon cells, maintaining the acidic environment of the intestine, inhibiting the formation of high levels of secondary bile acids, and promoting acidosis and apoptosis of cancer cells (33). Among them, butyric acid helps to balance proliferation, division and apoptosis of colon cells. Approximately 70%–90% of butyrate is produced by colon cell metabolism, and compared to healthy people, there is an obvious reduction in this type of acid in the stool of patients with colorectal cancer (34). Although SCFAs are derived from the intestinal flora, due to individual differences, the amount produced may not be sufficient to inhibit the development of colorectal cancer. Therefore, the consumption of probiotics can help increase the daily production of SCFAs. The presence of SCFAs can inhibit the growth of pathogens. In *in vitro* experiments, propionic acid and butyric acid inhibited expression of invasive genes encoded by Salmonella typhimurium, thereby preventing its attack on healthy cells (35).

**Figure 3:**
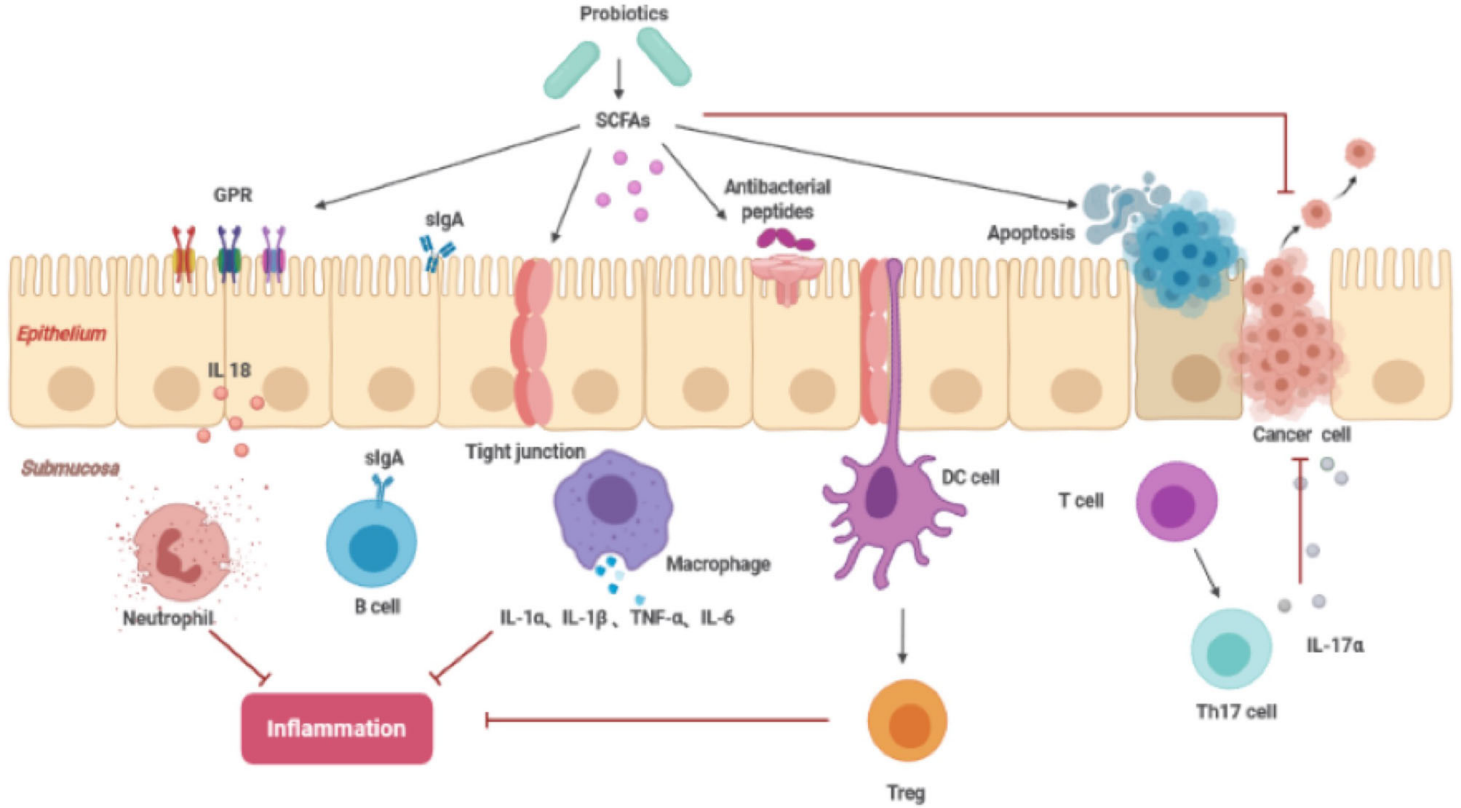
The function of SCFAs inhibiting cancer (SCFAs, short-chain fatty acids; sIgA, soluble IgA; GPR, G protein coupled free fatty acid receptor; DC cell, dendritic cell; Treg cell, regulatory T cell; Th17, T helper cell 17).

In addition, SCFAs can also regulate local intestinal immunity and the systemic immune response. SCFAs induce intestinal epithelial cells to produce antibacterial peptides and enhance the expression of tight junctions to stabilize intestinal barrier function. SCFAs affect inflammation by interacting with G protein-coupled receptors in the intestine and balancing the immune response (36). Conjugated linoleic acid (CLA) is an isomer of linoleic acid (LA), and both isomers can induce expression of apoptosis genes, including Bcl-2, caspase 3, and caspase 9, inhibiting the spread of colon cancer cells (**Figure 3**). Previous studies have reported that Lactobacillus, Bifidobacterium, Streptococcus salivarius, and Propionibacterium freudenreichii subspecies can produce CLA in the terminal ileum, which can be absorbed by colonic cells or interact with it to exert its beneficial effects (31).

These specific microbial strains can be used either alone or in combination with cancer treatment agents. The goal of treatment was achieved by activating immune surveillance against cancer (19). For example, Shi L et al. found that combined treatment with TGF-β receptor blockers and probiotics could enhance the antitumor immune response, thereby inhibiting the growth of tumors (37).

**Figure 4:**
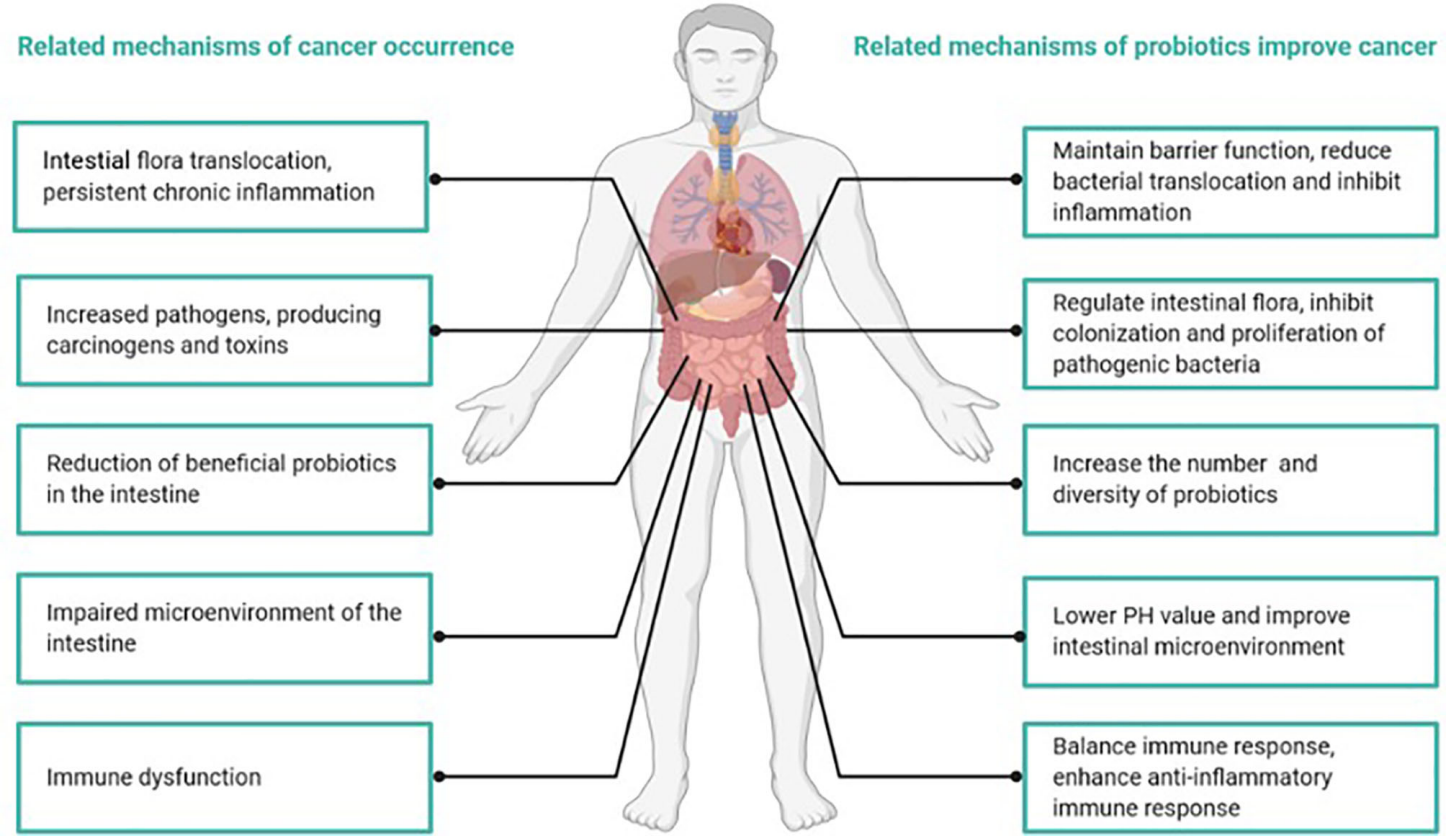
Mechanisms of cancer occurrence and how probiotics attenuate cancer.

Studies have indicated that the anticancer mechanisms of probiotics primarily include positive regulation of intestinal flora, changes in metabolic activity, the binding and degradation of carcinogenic compounds, immunomodulation to improve chronic inflammation, lowering intestinal pH and the inhibition of enzymes that produce potential carcinogenic compounds (26, 38) (**Figure 4**). The positive role of probiotics in the treatment of tumors has been confirmed, at least in animal models (39, 40).

Abnormal composition of the intestinal flora is a high-risk factor for colorectal cancer (41). The intestinal flora of patients with colorectal cancer usually contains a greater proportion of bacteria that cause gastrointestinal inflammatory diseases and bacteria that can produce toxins and carcinogenic metabolites (42). In contrast, SCFA-producing bacteria and potentially beneficial probiotics exhibit a decreasing trend (26). Chronic inflammation can make individuals susceptible to cancer (26). Studies found that under the mucus layer of the colon, Clostridium spp. were in direct contact with colon cells, invading the submucosa of the colon and causing persistent local inflammation (38). In addition, increased Clostridium spp. were found in colorectal cancer tissues, and they exhibited a profile of inflammation-related genes and proteins, such as COX-2, NF-κB, TNF-α, IL-6, IL-8 and IL-12, and matrix metalloproteinases 3 and 9, all of which contributed to tumor occurrence and transfer (26). Chandel D et al. found that the use of Lactobacillus rhamnosus GG, Lactobacillus acidophilus, or combination with celecoxib in a colorectal cancer animal model reduced NF-κB, COX-2, β-catenin, and K-ras carcinogenic biomarkers (43).

Compared to noncancer patients, the microbial structure of sample tissues from colorectal cancer patients was significantly different, and the diversity was lower (43). Treatment with probiotics increased the number and diversity of mucosal microorganisms and improved microbial structure (44). Pyrosequencing also revealed that probiotics could significantly reduce the abundance of the Fusibacter genus, which was previously suggested to be a contributing factor to tumorigenesis (45). Another preclinical study claimed that Bifidobacterium bifidum and L. acidophilus could be used as biotherapeutic agents to inhibit colon cancer by modifying intestinal bacteria (39). In people who are highly susceptible to colorectal cancer, probiotics might be used as an alternative biological therapy to prevent or even treat cancer (39).

In addition to gastrointestinal tumors, abnormal changes in the composition and function of intestinal microbes could also affect nongastrointestinal tumors, including liver cancer, pancreatic cancer and even breast cancer (25, 46). Through the portal venous system, the liver is uniquely exposed to intestinal bacteria and their metabolites, which may cause inflammatory changes and hepatotoxicity and ultimately directly lead to cancer. It has also been widely recognized that disturbance of the intestinal flora may cause liver cancer (47). For example, Hemophilus is a common pathogenic bacterium colonizing the colonic mucosa that has also been detected in human liver cancer tissues (47). Studies indicate that Hemophilus produces a lethal dilatant toxin after translocation to the liver and activates Wnt/β-catenin, NF-κB, p21, and Ki67 signaling in liver cells to induce liver cancer (19).

By constructing a mouse liver cancer model, Li J et al. confirmed that treatment with the probiotic E. coli Nissle 1917 enhanced the antitumor immune response, inhibiting tumor progression (40). The specific mechanism included Th17 cells and their product IL-17 being reduced in tumor tissues, while differentiation of Treg/Tr1 cells was enhanced, which affected expression of vascular growth factors and suppressed the progression of liver tumors through inflammatory and angiogenic mechanisms (40).

A recent study by Le Noci V et al. showed that probiotic aerosol therapy was beneficial for inhibiting lung melanoma metastasis (48). The lung microenvironment has high immune tolerance, and this feature prevents excessive inflammation caused by inhaled air particles (49). However, it also provides conditions for lung metastasis of various tumors (49). Lactobacillus rhamnosus induces the maturation of resident antigen-presenting cells, further activating lung T cells and NK cells and improving the immune suppression state, enhancing the antitumor immune effect (48). When used in combination with the chemotherapeutic drug dacarbazine, treatment efficacy was significantly enhanced. Probiotic aerosol therapy has become a new clinical therapy to prevent lung metastasis in high-risk melanoma patients (48).

Abnormal intestinal flora not only affects the pathogenesis of cancer but also participates in the therapeutic effect of anticancer treatment. Research in the past two years has emphasized the relationship between the microbiome and immunotherapy based on immune checkpoint inhibitors, such as PD-1/PD-L1 (50). Several research teams have discovered that the number, type and composition of the intestinal flora of cancer patients are closely related to the efficacy and survival of patients receiving PD-1 inhibitor therapy. The possible mechanism is that the interacting flora participates in the anticancer natural immune response (51, 52).

Routy et al. reported the response of patients with lung cancer, kidney cancer and bladder cancer to immunotherapeutic PD-1 blockade. They found that if patients had used broad-spectrum antibiotics before and after immunotherapy (two months before treatment and one month after the start of treatment), the bacteria in the body, including the intestinal flora, were disordered, and the immunotherapy effect was very poor. Both progression-free survival and the overall survival were significantly lower compared to patients who did not use broad-spectrum antibiotics. The bacteria Akkermansia muciniphila, enriched in the intestine, was the reason for some patients responding to PD-1 blockade (53).

In another clinical study, Gopalakrishnan et al. also found that the response of melanoma patients to anti-PD1 immunotherapy was related to the diversity and composition of trillions of beneficial and harmful bacteria in the digestive tract. Based on the analysis of patient stool samples, it was found that compared to patients who did not respond to PD1 checkpoint inhibitor treatment, patients who did respond to PD1 checkpoint inhibitor treatment had a more diverse intestinal flora, and the content of Clostridium order was increased. There are a large number of Bacteroides bacteria in the intestine of melanoma patients who have not responded to treatment, and their bacterial diversity is far less than that of melanoma patients who have responded to treatment. By detecting the presence of important immune system cells in the patient’s tumor, patients who respond to anti-PD1 immunotherapy were found to have higher levels of immune infiltration, including CD8^+^ killer T cells related to specific bacteria (52).

The intestinal flora is not a necessary condition for the antitumor effect of chemotherapeutic drugs, and experiments have found that the survival rate of sterile or flora-depleted mice was significantly reduced (52). After treatment with lactic acid bacteria, the anticancer effect of chemotherapeutic drugs was restored. These results indicate that the flora might facilitate the chemotherapy effect through a flora-dependent mechanism (54).

In conclusion, *in vitro* studies have found that probiotics induce tumor cell apoptosis and inhibit tumor cell proliferation and metastasis. In animal models, probiotics improve tumor conditions. This positive effect provides a basis for clinical trials. However, considering that most of the current research on probiotics and cancer is limited to gastrointestinal tumors, the specific mechanism of probiotics against tumors has not been fully elucidated. Even in animal experiments, because most tumors are induced by chemical drugs, they are different from the complex pathogenesis of human tumors, so the therapeutic effects of probiotics must be carefully considered.

## The Role of Probiotics in the Treatment of Antitumor Side Effects

Gastrointestinal discomfort is a common side effect of antitumor therapy. Radiochemotherapy directly kills intestinal cells, and the stress response it causes leads to destruction of the intestinal mucosal barrier. In the case of increased permeability of the intestinal mucosa, intestinal flora and endotoxins enter extraintestinal tissues and organs, causing uncontrolled systemic inflammation and multiple organ failure (55, 56). Surgery may result in impaired physiological gastrointestinal function. Diarrhea can be caused by a significant reduction in the transit time of food through the intestines and excessive bacterial growth (57). Antibiotics are often used during treatment, which can also affect the microbiome (58). Probiotics based on Bifidobacterium and Lactobacillus can effectively resist the growth of harmful bacteria through biological action (59). Supplementing with probiotics can improve the intestinal environment, enhance intestinal mucosal barrier function, and reduce the occurrence of diarrhea (57, 59). Recent studies revealed that the improvement of antitumor side effects by probiotics was also related to innate immunity. For example, probiotic cell wall acyl dipeptides alleviate mucosal damage caused by antibiotic chemotherapeutics by stimulating intracellular pattern recognition receptors (NOD2) (57, 59). In general, probiotics may have a beneficial effect by improving diarrhea caused by radiochemotherapy or surgery and rarely cause side effects.

In addition to restoring the intestinal mucosal barrier, probiotics can also attenuate oral mucosal damage induced by chemotherapy. In clinical treatment, more than 70% of hematological patients receiving high-dose chemotherapy and hematopoietic stem cell transplantation (HSCT) may develop grade III or IV oral mucositis, which causes great pain. Atul Sharma et al. analyzed the efficacy of Lactobacillus CD2 in preventing grade III/IV mucositis in patients receiving HSCT (60). Only 19% of patients developed grade III or IV mucositis. The median time to onset and recovery were 6 days and 8 days, and throughout the observation process, no adverse reactions related to probiotics were observed (60).

Probiotics also help in systemic inflammation, such as graft-versus-host disease (GVHD). Donor-derived T cells, proinflammatory cytokines, and LPS are the primary triggers of GVHD, in which the intestine is one of the organs most affected by GVHD and a key determinant of GVHD severity. The occurrence of GVHD greatly limits the feasibility and efficacy of HSCT (61). An intact intestinal barrier plays an important role in the development of GVHD, and LPS can enter the circulatory system through the damaged mucosal barrier to induce GVHD (62). In animal experiments, oral administration of L. rhamnosus GG before and after transplantation improved the survival rate of mice, especially between 7 and 14 days after transplantation, and the reduction in mortality was even more pronounced (63). Probiotic administration in patients receiving HSCT may also reduce the incidence of stage III-IV acute GVHD. One ongoing study showed that probiotic supplementation therapy reduced the bacterial translocation of mesenteric lymphoid tissue and the reduction of terminal ileal histological inflammation, indicating that probiotics can indeed attenuate GVHD (64).

Emerging data indicate that there is a strong correlation between abnormal microbiota composition and intestinal manifestations of acute GVHD (65). Although it has been observed that probiotics can improve GVHD in animal models, the mechanism is poorly understood. There are reports that SCFAs directly act on intestinal epithelial cells to promote recovery (65). Studies have also shown that IL-22 plays an important role in mediating the recovery of intestinal stem cells in GVHD, which might be related to its function of promoting Paneth cells to secrete antimicrobial peptides and mediating epithelial regeneration (65).

Similarly, probiotic metabolites may also ameliorate GVHD. Indole or indole derivatives metabolized by tryptophan in the intestinal flora can limit intestinal inflammation caused by various stressors (66). Indole-3-carbaldehyde (ICA), an indole derivative, reduced intestinal bacterial translocation and inflammatory cytokine production in mice through type I IFN signaling (66). In mice lacking type I IFN signaling, the protective effect of ICA was eliminated after radiation exposure (66). These data indicate that indole could assist in limiting acute GVHD-related damage while retaining the antitumor response (66). In general, intestinal GVHD is characterized by the destruction of the integrity of the intestinal epithelial barrier and the disorder of flora. Therefore, probiotics and their production, which remodel the microbial community, inhibit pathogens, reduce inflammation and restore the intestinal epithelial barrier, might represent a good treatment strategy for GVHD in the future (67).

Compared to the lack of clinical data for probiotics to treat tumors, there are more clinical trial results demonstrating that probiotics have certain benefits in attenuating antitumor-related side effects (**Table 1**).

**Table 1:** Clinical trials using probiotics to improve the side effects of anticancer therapy.

Malignancy	Case number	Treatment strategy	Objective	Intervention	Outcome	Side-effect	Reference
Cervical cancer	54	Radiotherapy	Improve diarrhea	From day 1 to the end of radiotherapy, receive 3 capsules per day, each containing 1.75 billion live bacteria (Lactobacillus acidophilus LA-5 and Bifidobacterium animalis subsp. BB-12)	The incidence of diarrhea in the probiotic group was lower than placebo group (53.8 and 82.1%, p <0.05), and the use rate of the anti-diarrhea drug loperamide was significantly reduced (p <0.01)	No probiotics-related toxicity reported	(46)
Colorectal cancer	150	Postoperative chemotherapy	Improve diarrhea	1-2×10^10^Lactobacillus rhamnosus GG supplements daily	Patients receiving probiotics had mild diarrhea, and the incidence of grade 3 or 4 diarrhea (experimental group vs control group: 22% vs 37%, P = 0.027)	No probiotics-related toxicity reported	(45)
Lungcancer	41	Chemotherapy	Improve diarrhea	Starting one day before chemotherapy, take C. butyrate 3 times a day (420 mg/tablet) for 3 weeks	The incidence of grade I diarrhea was lower in the probiotic group (20% and 42.86%)	No probiotics-related toxicity reported	(59)
Gastric cancer	120	Surgery	Improve diarrhea	Nutrient formula food rich in fiber and probiotics, providing enteral nutrition for 7 consecutive days after surgery	Diarrhea cases decreased in combination of fiber and probiotics group	No probiotics-related toxicity reported	(49)
Head and neck cancer	200	Radiotherapy and chemotherapy	Improve oral mucositis	From the first day of treatment to 1 week after the last treatment, Lactobacillus brevis CD2 tablets (not less than 2 × 10^9^), 6 times a day	The incidence of grade III and IV mucositis in the probiotics was lower than placebo group (52% and 77%, P <0.001), the completion rate of anticancer treatment in probiotic group was significantly improved (92% and 70%, P = 0.001)	No probiotics-related toxicity reported	(60)
Colorectal cancer	52	Surgery	Improve inflammation	Starting 4 weeks after surgery, oral administration of 30 billion probiotic mixed preparations twice a day for 6 months	Inflammatory cytokines in probiotics group were significantly reduced, including TNF-α, IL-6, IL-10, IL-12, IL-17A, IL-17C, and IL-22 (P<0.05)	No probiotics-related toxicity reported	(61)

## Safety Assessment of Probiotics

As additional supplementary active microorganisms, the adverse reactions of probiotics, primarily including systemic infections, gastrointestinal side effects, skin reaction, access to antibiotic resistance genes, harmful effects of probiotic metabolites and abnormal stimulation of the immune system, must be considered. The population at highest risk includes infants, the elderly, hospitalized patients, and patients with immunodeficiency due to genetic or acquired diseases (68). Studies have shown that the incidence of bacteremia in patients using yeast is approximately 1/5.6 million and for lactic acid bacteria is less than 1/1 million (69). The results of another large-scale epidemiological study indicated that infections caused by Lactobacillus and Bifidobacteria were extremely rare, accounting for 0.05%–0.4% of the total cases of infective endocarditis and bacteremia, and most patients had severe underlying diseases (70). In addition to being related to individual factors, the risk of infection was also related to the type and dose of the probiotics. It was reported that compared to Bifidobacterium, Lactobacillus was more likely to cause infection (71, 72).

One of the most important theoretical issues in the clinical use of probiotics is bacteremia, while fungal infections caused by yeast are even more difficult to treat. Compromised intestinal integrity and probiotic translocation are the main causes (73). Genomics data confirmed that these adverse reactions were indeed related to ingested probiotics rather than colonized probiotics in the intestine (74). It was found that for patients with impaired immune function, the risk of infection was far higher. Redman et al. conducted a systematic retrospective study and found that five of 1530 patients reported probiotic-related bacteremia, although probiotic management did indeed improve the severity and frequency of diarrhea in these cancer patients (75). Therefore, in cancer patients, the serious invasive disease caused by probiotics deserves vigilance (**Table 2**) (76–80).

**Table 2:** Five reported cases of probiotic-related bacteremia.

Age/Gender	Malignancy	Treatment strategy	Objective	Probiotics strains	Neutropenia	Side-effect	Outcome	Reference
8-month old baby	Acute myeloid leukemia	Intensive treatment with high-dose idarubicin, cytarabine, and etoposide (ICE)	Prevention of chemotherapy-related diarrhea	Saccharomyces boulardii	Yes	Saccharomyces cerevisiae strain isolated from blood culture	Anti-fungal treatment was performed for 14 days until full recovery from neutropenia. The patient eventually undergoes HLA-matched sibling donor bone marrow transplantation	(76)
65-year-old male	Oropharyngeal carcinoma, T3 N2 M0	Chemotherapy plus radiotherapy, including cisplatin and 5-fluorouracil plus external radiation (60 Gy)	Treat aseptic diarrhea	Saccharomyces boulardii	Not report	Saccharomyces cerevisiae strain found in blood culture	Amphotericin B, 60 mg/day for 4 weeks, fever decreased. Evaluation after 6 months showed partial remission of the tumor with no signs of residual infection	(77)
38-year-old male	Stage IV Hodgkin lymphoma with AIDS	Chemotherapy (specifically unknown)	Unknown	Lactobacillus acidophilus	Not report	Lactobacillus acidophilus found in blood culture	Clindamycin combined with gentamicin treatment, on day 3, blood culture was negative. On the 10th day, he was discharged from the hospital and received home care	(78)
69-year-old male	Stage IIIA mantle cell lymphoma	4 cycles of alternating Rituxan-Hyper CVAD Part A (rituximab, CL, VCR, doxorubicin and dexamethasone) and Part B (Ara-C and MTX) chemotherapy, after 4 months of chemotherapy, hematopoietic stem cell transplantation	Improve mucositis	6–8 cups of yogurt on the market rich in probiotics	Not report	Lactobacillus acidophilus found in blood culture	Antibiotic treatment (specifically unknown), symptoms relieved and discharged after 1 week	(79)
73-year-old male	Chronic lymphocytic leukemia	Unknown	Unknown	Bacillus subtilis	Not report	Bacillus subtilis found in blood culture	From day 1 to 16, imipenem treatment, day 16 later combined with antibiotic treatment (ceftazidime, amikacin and vancomycin) and intravenous immunoglobulin, fever quickly reduced. Death on day 25, may be due to central nervous system involvement	(80)

In another systematic retrospective study, currently managed probiotic strains (primarily Bifidobacterium and Lactobacillus), dosage (daily supplemental doses did not exceed 5.0 × 10^10^ CFU/day, median was 2.0 × 10^9^ CFU/day), and there were no serious adverse reactions caused by the probiotics. The results showed that it was safe to use probiotics in patients with impaired immune function, including very severe patients. However, most of the studies focused on the efficacy of probiotics rather than safety, and large-scale clinical studies are needed to further determine their true safety (81).

HSCT has become the standard treatment for many adult and childhood malignant tumor diseases, but the side effects caused by the treatment cannot be underestimated (82). Increasing evidence shows that the diversity of the microbiome is disturbed during treatment, often leading to abnormal systemic immune responses, pathogen colonization and mucosal invasion. There were also studies showing that the loss of microbial diversity was an independent risk factor for death after allogeneic HSCT (83).

Probiotics protect the microbiome and can minimize the risk of gut-mediated diseases. However, their safety has not been fully evaluated in the case of HSCT. Recently, Ladas et al. evaluated the safety and feasibility of probiotics in 30 children and adolescents who had undergone allogeneic HSCT (84). In the time range that coincided with intestinal mucosal damage and accompanying neutropenia, no cases of probiotic bacteremia (0% (0/30), 95% CI 0-12%) were observed, and there were no other unexpected adverse events. Although new infections of C. difficile were found in 20% of participants, studies confirmed that they were not related to probiotic management (84). Their research provides preliminary evidence that use of probiotics is safe and feasible in children and adolescents undergoing HSCT (84). Another study showed that for patients who received unrelated cord blood transplantation, early-stage yogurt supplementation was safe and feasible, and no unexpected adverse events caused by probiotics were observed (85). Therefore, in patients receiving HSCT, probiotics may have a positive role in maintaining the health of the intestinal flora and improving the patient prognosis.

However, in one clinical study, it was believed that probiotics did not benefit patients with acute myeloid leukemia undergoing intensive treatment or bone marrow transplantation (86). Instead, the probiotic treatment group exhibited a higher incidence of infection, especially blood infection (86). The researchers concluded that in patients with a long-term risk of neutropenia, without other indications for using probiotics, it was not recommended for such patients to use probiotics (86).

## Conclusions

As a dietary supplement, probiotics lack strict standards for efficacy and safety certification. Although the efficacy of several strains has been experimentally supported, the health-promoting effects of most probiotics have not been proven. Relevant publicity of probiotic products rarely mentions the potential risks.

In a number of trials evaluating the protective effects of probiotic therapy on antitumor treatment-related side effects, combined use of probiotic strains did have a positive protective effect for patients with respect to certain immune functions (47). However, for patients with severely impaired immune function, especially patients with neutropenia, careful consideration is required (87). Due to the complex pathogenesis of tumors, different patients receive different treatment options, and different strains will affect the results, so large-scale clinical trials are urgently needed.

Identifying the most beneficial strains for the prevention and treatment of different types of cancer requires a very extensive human database, and it is necessary to carefully analyze correlations between different strains and clinical responses. Once we have identified a beneficial flora for cancer prevention and treatment, the next challenge is how to use probiotics and their products to regulate patient flora. At the same time, we can use the intestinal flora as a new cancer biomarker based on its response to changes in the pathophysiological environment. The ultimate goal is to identify specific strains or combinations of strains that can both reduce the side effects of cancer treatment and boost anticancer treatment (88). Therefore, for cancer and other diseases, the regulation of targeted human flora is likely to become a new field of precision and personalized medicine in the future.

## Author Contributions 

KL and SD prepared the original draft. XW and RJ reviewed and edited the draft. HC supervised and finalized the manuscript. All authors contributed to the article and approved the submitted version.

## Funding

This research was funded by National Natural Science Foundation of China (No. 31701207 to HC).

## Conflict of Interest

The authors declare that the research was conducted in the absence of any commercial or financial relationships that could be construed as a potential conflict of interest.
